# Transcranial and pulsed focused ultrasound that activates brain can accelerate remyelination in a mouse model of multiple sclerosis

**DOI:** 10.1186/s40349-018-0119-1

**Published:** 2018-12-10

**Authors:** T. A. Olmstead, P. A. Chiarelli, D. J. Griggs, A. M. McClintic, A. N. Myroniv, P. D. Mourad

**Affiliations:** 1 Department of Neurological Surgery, University of Washington, Seattle, WA 98195 USA; 2 Division of Engineering and Mathematics, University of Washington, Bothell, WA 98011 USA

**Keywords:** Focused ultrasound, Neuromodulation, Brain activation, Multiple sclerosis, Therapy

## Abstract

**Background:**

Multiple sclerosis (MS) impacts approximately 400,000 in the United States and is the leading cause of disability among young to middle aged people in the developed world. Characteristic of this disease, myelin within generally focal volumes of brain tissue wastes away under an autoimmune assault, either inexorably or through a cycle of demyelination and remyelination. This centrally located damage produces central and peripheral symptoms tied to the portion of brain within the MS lesion site. Interestingly, Gibson and colleagues noted that optical activation of transgenically tagged central neurons increased the thickness of the myelin sheath around those neurons. Since ultrasound, delivered transcranially, can also activate brain focally, we hypothesized that ultrasound stimulation that followed the temporal pattern of Gibson et al. applied to MS lesions in a mouse model might either decelerate the demyelination phase or accelerate its remyelination phase.

**Methods:**

We created a temporal pattern of ultrasound delivery that conformed to that of Gibson et al. and capable of activating mouse brain. We then applied ultrasound, transcranially, following that temporal pattern to separate cohorts of a mouse model of multiple sclerosis, using three different ultrasound carrier frequencies (0.625 MHz, 1.09 MHz, 2.0 MHz), during each of the demyelinating and remyelinating phases. After identifying the most promising protocol and MS brain state through qualitative analysis of myelin content, we performed additional studies for that condition then assayed for change in myelin content via quantitative analysis.

**Results:**

We identified one ultrasound protocol that significantly accelerated remyelination, without damage, as demonstrated with histological analysis.

**Conclusion:**

MRI-guided focused ultrasound systems exist that can, in principle, deliver the ultrasound protocol we successfully tested here. In addition, MRI, as the clinical gold standard, can readily identify MS lesions. Given the relatively low intensity values of our ultrasound protocol – close to FDA limits – we anticipate that future success with this approach to MS therapy as tested using more realistic MS mouse models may one day translate to clinical trials that help address this devastating disease.

## Introduction

Multiple sclerosis (MS) represents the leading cause of disability among young to middle aged people in the developed world [1], with over 400,000 people in the United States affected by it [2]. Its characteristic loss of central and peripheral function arises due to an autoimmune assault on the myelin in the brain, often with an initial ‘relapsing-remitting’ phase (with attendant loss and at least partial recovery of function) followed by net degradation of myelin and function [2].

Interestingly, a range of studies show that the quality and quantity of myelin around a given axon depends upon the activation of that axon [3, 4]. Using a disease-free optogenetic mouse model, a more recent study demonstrated enhanced myelin buildup around axons activated by pulsed laser light delivered via fiber optic cable with a specific temporal pattern [5]. Finally, several studies show that transcranial delivery of pulsed focused ultrasound (pFU) can non-destructively activate central neural circuits across a range of species, including mice [6], rats [7], rabbits [8], sheep [9], non-human primates [10] and humans [11], reviewed recently in a comprehensive fashion [12].

Taken together, these citations motivated the hypothesis governing the present work: targeted transcranial pFU stimulation of axons within MS lesions in a manner that follows the laser-light temporal pattern of Gibson et al. (2014) - (reference [5]) - can decrease these lesion’s rate of demyelination and/or increase their rate of remyelination.

To test this hypothesis, we applied three pFU protocols, all with a temporal pattern that conformed to that delivered by Gibson et al., each with a different carrier frequency, and all with relatively low ultrasound intensity values, to one side of the brains of separate sets of a mouse model of MS. One set experienced chemically induced demyelination; the other set experienced remyelination after cessation of chemically induced demyelination. We used histopathological measures of myelin to determine the effect of pFU neural activation on myelin content, with the contralateral hemisphere serving as a control. We also stained for neuronal structure, to assay for possible damage created by the ultrasound.

## Methods

### Animal model

All animal procedures were approved by the University of Washington Institutional Animal Care and Use Committee under protocol #4084–08 and conformed to applicable national guidelines.

Adult male C57BL/6 J mice weighing approximately 25-30 g (The Jackson Laboratory, Bar Harbor, ME) were used for all procedures.

**Fig. 1:**
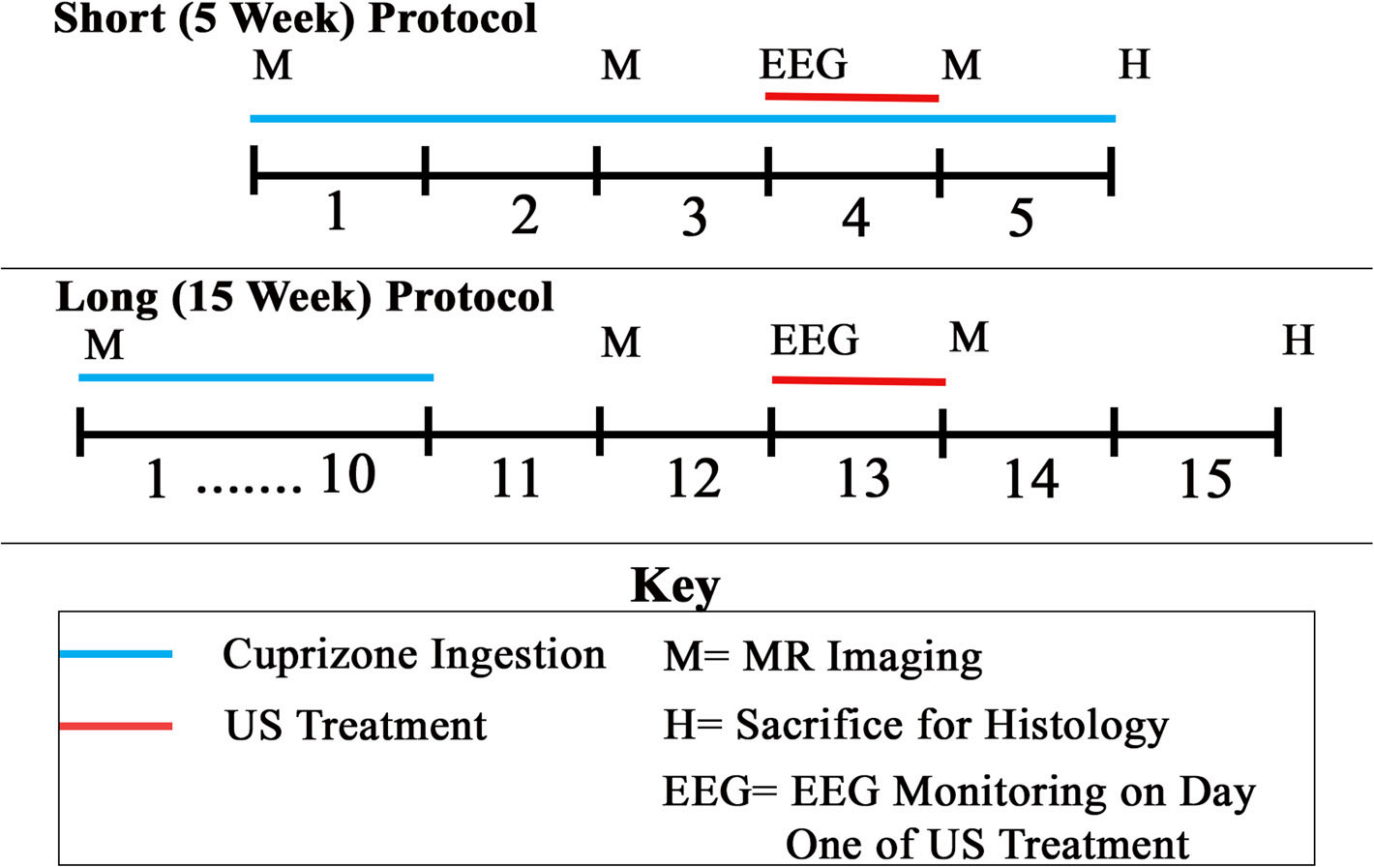
Map of the time course of the experimental procedures. We used two experimental protocols: a ‘short, 5 week’ protocol that tested ultrasound’s effect on demyelination and a ‘long, 15 week’ protocol that tested ultrasound’s effect on remyelination. Both protocols required ultrasound application every day for a week, when appropriate. They differed by the timing of application of Cuprizone chow, ultrasound treatment, MRI imaging and EEG monitoring

Mice were fed a 0.2% Cuprizone diet (Envigo, Madison WI) following two separate feeding protocols, described below. The Cuprizone mouse model of demyelination produces multiple sclerosis-like reduction in myelin primarily in the corpus callosum and superior cerebellar peduncles [13–16]. These effects are visible via T_2_-weighted MRI after 4 weeks of Cuprizone administration [14], with full demyelination occurring by 6 weeks [17]. Cuprizone-induced demyelination can switch to spontaneous remyelination as early as 4 days after Cuprizone withdrawal [16], making it convenient for studying therapies that may prevent demyelination as well as accelerate remyelination [18]. We used two separate protocols for Cuprizone administration (Fig. 1). For the first, short, 5-week protocol, mice received Cuprizone chow for the entire 5 weeks, which generated demyelination. For the second, long, 15-week protocol, mice received Cuprizone chow for the first 10 weeks then reverted to standard chow, thereby allowing remyelination after 10 weeks. With the 5-week protocol we sought to test the ability of pFU-activation of brain to slow demyelination. With the 15-week protocol we sought to test the ability of pFU-activation of brain to increase the rate of remyelination.

### Ultrasound application – General considerations

**Fig. 2:**
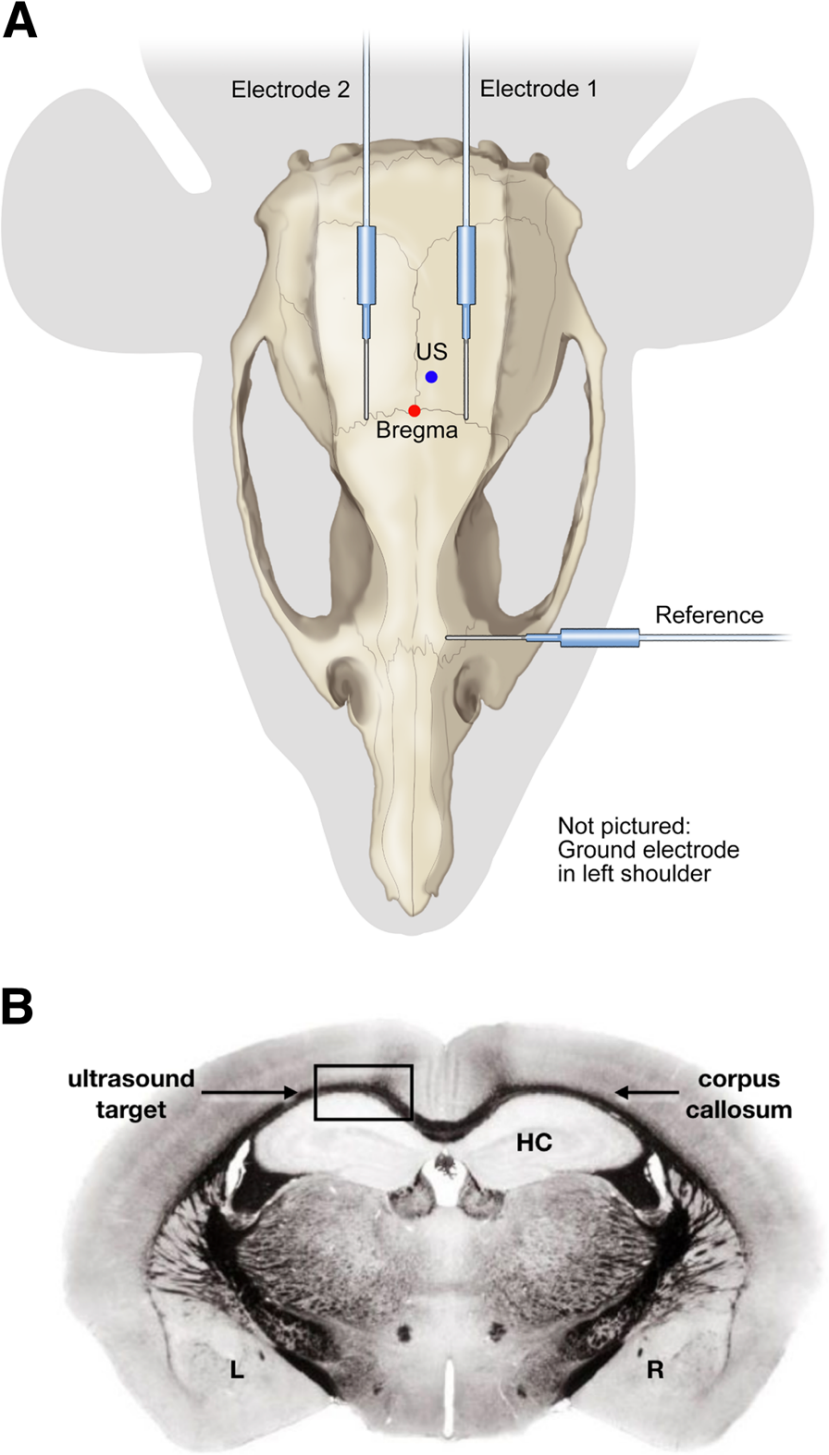
Global view of the experiment. **a** Plan view. We delivered ultrasound transcutaneously and transcranially to the left side of mouse brain, using a micro-positioner to deliver that ultrasound to − 1.03 mm behind Bregma, 0.5 mm to the right of center, and 0.5 mm below the skull – within the corpus callosum, below the somatosensory cortex and above the hippocampus. We placed two subdermal electrodes on either side of the ultrasound focus. We placed a reference electrode rostral to the site of ultrasound application, and a grounding electrode subcutaneously on the mouse’s shoulder. **b** Coronal view. This schematic shows a coronal view of mouse brain, stained for myelin, at − 1.03 mm Bregma, highlighting the corpus callosum (the ‘eye brow-like’ black and curved line), the hippocampus (HC) and the point of ultrasound exposure

Ultrasound was applied (with instruments, protocols and time points described below) while animals were under anesthesia as follows: we induced an initial deep anesthesia through a injection of a mixture of ketamine (86.7 mg/Kg) and xylazine (9.3 mg/Kg), with supplemental doses administered as needed to maintain an even anesthetic plane. Toe pinches were used to check depth of anesthesia. A circulating water heating pad (Gaymar Industries, Orchard Park, NY) set to 100 ° F maintained animal body temperature during the procedure. After induction of anesthesia, we placed the mice in a stereotaxic headpiece. To facilitate ultrasound transmission through the skull, hair was removed from the top of each mouse’s head using shears and depilatory cream. Through use of a sub-millimeter resolution micro-positioner, we placed the ultrasound’s focus in the left hemisphere of the mouse’s brain such that it overlapped with the center of the corpus callosum, itself under the somatosensory cortex and above the hippocampus – Fig. 2.

### Ultrasound sources

We used three different transducers for pFU-based brain activation, depending upon the specific study as described below, with each choice motivated by existing ultrasound systems, two of which are ready now for application to human brain. For the lowest ultrasound frequency, we used a custom-made focused ultrasound transducer with center frequency 0.625 MHz and accompanying matching network (Applied Physics Laboratory, University of Washington, Seattle WA), consistent with the Insightec ExAblate MRI-guided ultrasound device currently available for treatment of brain [19, 20]. Our mid-frequency system had a carrier frequency of 1.09 MHz with an accompanying matching network (Model Number H − 101, Sonic Concepts, Woodinville, WA), close to the 1.0 MHz carrier frequency of the Sonalleve MRI-guided therapeutic ultrasound device, developed by Philips Ultrasound, now held by Profound Medical [21] and another, similar MRI-guided therapeutic device developed by Imasonic [22]. We also used a higher-frequency focused ultrasound transducer, with a carrier frequency of 2.0 MHz (Model Number H − 148 Annular Array, Sonic Concepts, Woodinville, WA), consistent with existing transcranial Doppler devices, hence capable of transcranial delivery though currently without MRI guidance. We used a calibrated needle hydrophone (HNR-1000, Onda Corporation, Sunnyvale, CA) placed in a tank filled with deionized, degassed water to calibrate all transducers. We drove our transducers using an appropriate arrangement [7] of three function generators (HP33120A, Hewlett-Packard, Palo Alto, CA, USA), an oscilloscope (Wave Runner LT 322 oscilloscope, LeCroy Corporation, Chestnut Ridge, NY, USA), and an amplifier (ENI Model A150 RF Power Amplifier, ENI, Rochester, NY).

### Ultrasound and Cuprizone protocol during demyelination (short, 5-week protocol)

Cohorts of 3–4 mice received Cuprizone chow for a total of 5 weeks, without reverting to standard chow (again, Fig. 1). pFU was applied for five consecutive days during the fourth week of Cuprizone treatment before complete demyelination in an attempt to decelerate or even reverse demyelination. Following this therapeutic protocol, the mice recovered for 2 weeks before euthanasia to facilitate histology.

### Ultrasound and Cuprizone protocol during remyelination studies (long, 15-week protocol)

Cohorts of 3–4 mice received Cuprizone chow for 10 weeks (Fig. 1). After 10 weeks, animals were then fed standard chow, subsequently experiencing remyelination. After an additional 2 weeks, hence during week 13, pFU was applied to the mice each day for 5 days in order to test the ability of pFU to accelerate remyelination. Following this therapeutic protocol, the mice recovered for 2 weeks before euthanasia to facilitate histology.

### Ultrasound pulse sequence and intensity for 5- and 15-week protocols

**Fig. 3:**
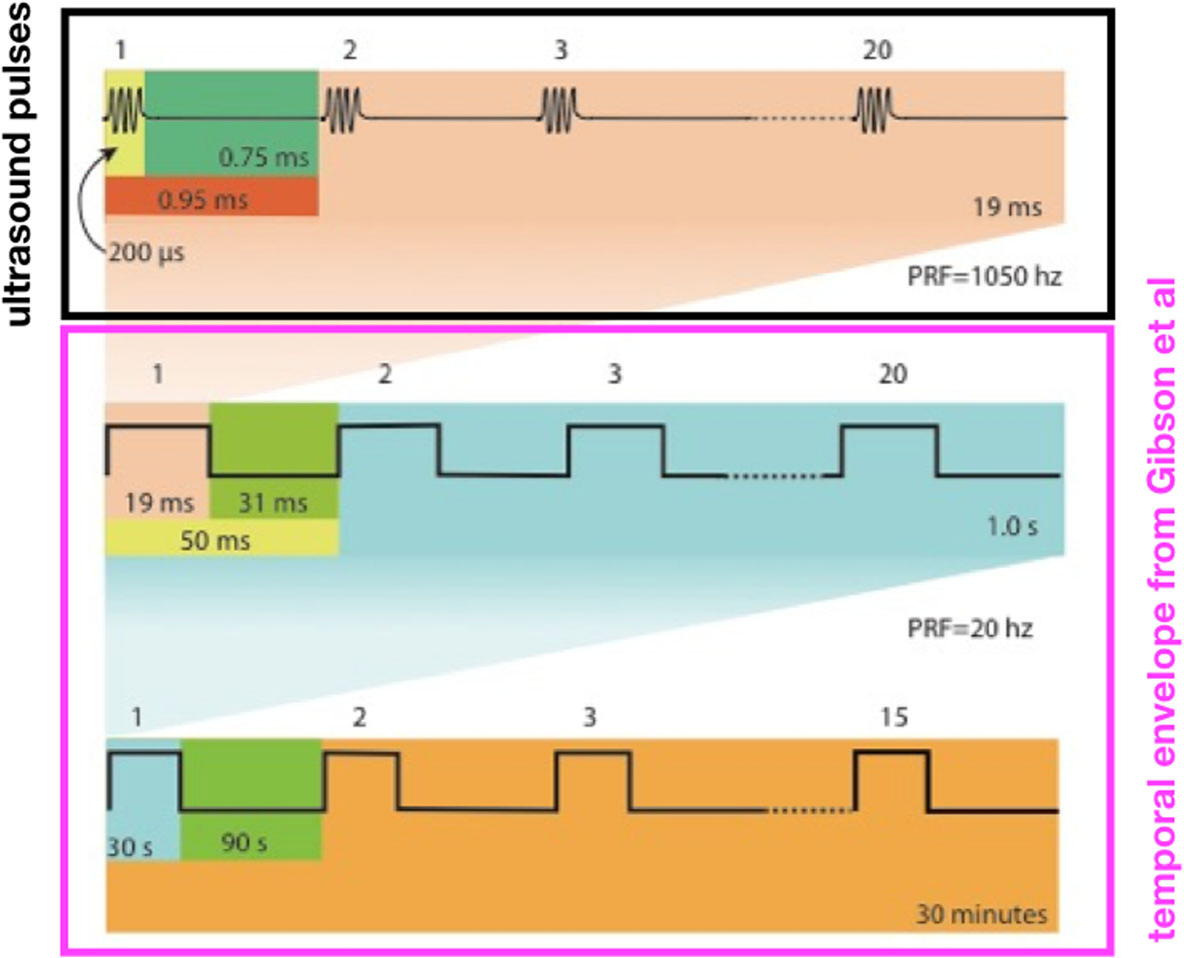
Temporal pattern of ultrasound delivery, modeled after the optical stimulation pattern of Gibson et al. The optical protocol delivered light twenty times per second, with each pulse of light lasting 25 milliseconds (ms), for a total of 30 s. Each 30-s long period of pulsed stimulation preceded a 90-s long period of no stimulation. These on-off periods of light exposure lasted a total of 30 min per day, applied for five contiguous days. We modified this protocol to application of ultrasound by using *pulsed* ultrasound during *each* of 19-ms long time periods, followed by 31 ms of no ultrasound, rather than *continual* light for 25 ms followed by 25 ms of no light. The figure here shows 20 ultrasound pulses applied during each 19 ms time period, each pulse lasting 200 microseconds

Figure 3 shows the ultrasound application pattern, whose temporal envelope mimics that of Gibson et al. (2014). Specifically, that envelope consists of 20 stimulations per second lasting for 30 s, followed by a rest period of 90 s, repeated for a total of 30 min per day, for each of 5 days.

Note that Gibson et al. applied *continual* light pulses, each lasting 25 milliseconds (ms) and followed by no light for 25 ms, 20 times per second. While continually applied ultrasound can activate brain [23], we followed the majority of papers (reviewed recently in Bobola et al. [12]) as well as our own work [7, 24], by stimulating brain with a set of rapidly applied ultrasound bursts for each of the 20 times per second within the Gibson et al. protocol. Specifically, 20 times per second, we applied a set of 20 very short pulses of ultrasound (each lasting 200 microseconds) at a pulse repetition frequency (PRF) of 1050 Hz. This ultrasound stimulation burst lasted for 19 ms followed by 31 ms of no light (Fig. 3, top row). Each ultrasound stimulation burst was then repeated at 20 Hz for 30 s (30 s of pulsed ultrasound “on”), with a rest-period of 90 s of no ultrasound (90 s of ultrasound “off”) – Fig. 3, middle and bottom row. This continued for 30 min per ultrasound session, with one session per day over a five-day period. This ultrasound scheme was used for each of the 5- and 15-week experimental protocols.

With regard to intensity, we performed exploratory studies with naive mice to identify a single ultrasound intensity value that produced reliable brain activation across all three ultrasound carrier frequencies. That analysis yielded ultrasound with a spatial peak intensity averaged over each 200 microsecond long pulse of 1.52 W/cm^2^ (I_sppa_). Twenty of them applied over 19 milliseconds (a single ultrasound burst) had a spatial peak burst average intensity of 3.2 W/cm^2^ (I_spba_) or, averaged over time instead of over a burst, 0.06 W/cm^2^, spatial peak temporal average intensity (I_spta_). With 20 such ultrasound stimulation bursts per second, we therefore used 1.2 W/cm^2^ (I_spta_) within the complex temporal pattern shown in Fig. 3, not far above the FDA guideline for diagnostic ultrasound [25] of 0.72 W/cm^2^ (I_spta_) and consistent with intensity values applied to anesthetized rats that changed their response to anesthesia [26].

### EEG monitoring

We monitored brain activity using subdermal electrodes (with details below) on only the first of 5 days of ultrasound application. In this way we minimized discomfort for the animal and avoiding implanted electrodes, which would have interfered with the MRI imaging. During a given EEG session, time-series recordings were first obtained for 4 min, without any ultrasound – the baseline recording. Next came application of ultrasound for 30 min, with the specific temporal pattern as described in Fig. 3. For each 30-min EEG recording corresponding to a single ultrasound protocol, the time-series was dissected into 50 millisecond (ms) windows during which time ultrasound was on during the first 19 ms, and off during the remaining 31 ms. We designate that second, off period as ‘background’.

We used two subdermal EEG electrodes, arranged in a single-channel bipolar configuration, with a third electrode for reference and a fourth for ground. Figure 2a shows the locations of the subdermal needle electrodes and their relation to landmarks on a mouse skull. Our EEG setup used thin wire EEG electrodes (Ambu Neuroline Subdermal 27G, Cadwell, Kennewick, WA) recorded at a sampling rate of 38,400 Hz on a 16-channel biosignal amplifier (gUSBamp, Guger Technologies OG, Graz Austria) that integrated with Simulink (MathWorks, Natick MA). EEG monitoring and analysis was performed during ultrasound application. EEG results were analyzed using MATLAB software (MathWorks, Natick MA) and reported as an average plus standard error over each 19 millisecond pulses over the entire time of ultrasound application, a total of {20 bursts/s} X {30 s/[2 min]} X {30 min/day} X {5 days of ultrasound application} = 45,000 bursts.

### Magnetic resonance imaging (MRI)

T_2_- weighted baseline MRI scans were obtained before administering Cuprizone on the first of the first week of both the 5- and 15-week MS protocols. Scans were also collected during the week before pFU treatment (pre-US), and the week after pFU treatment (post-US), as represented in Fig. 1. All scans were conducted at the University of Washington High Resolution Imaging (HRIM) facility using a 14 Tesla (600 MHz) vertical wide bore Bruker Magnetic Resonance Spectrometer (Bruker Corporation, Billerica MA) with integral temperature control and isoflurane anesthesia capability. Resultant DICOM image files were converted to standard image formats (tiff) using Adobe Photoshop CC 2017 (Adobe Systems Incorporated, San Jose CA). T2 parameters follow that of Thiessen et al. (2013) - (reference [14]).

### Histology and image analysis

At the completion of the study, all 5- and 15-week MS protocol animals were transcardially perfused in 10% Neutral Buffered Formalin (Fisher Protocol, Fisher Scientific, Hampton NH) after induction of deep anesthesia with isoflurane. Following perfusion, animals were decapitated, and heads were post-fixed in Formalin for at least 1 week before brain removal for sectioning and staining.

#### Histological staining

After sufficient fixation, the brains were sectioned at 12 μm, and mounted onto Superfrost Plus microscope slides (Fisherbrand, Fisher Scientific, Hampton NH). These slides were then stained, one stain per slide, with Luxol fast blue for myelin, or Cresyl Violet (a neuronal stain) to assay for cellular damage.

#### Histological image acquisition

Histological images were taken using a Nikon Eclipse TE200 inverted microscope with a Nikon DS-Fi1 camera, a Nikon 0.6x TV lens, and a 4x magnification objective. The images were collected in color using unfiltered bright-field settings. Due to the method in which sections were collected and mounted, the left side of each slide corresponds to the left side of the resultant image, and also corresponds to the left side of the animal itself, where we applied ultrasound.

#### Histological image analysis

**Fig. 4:**
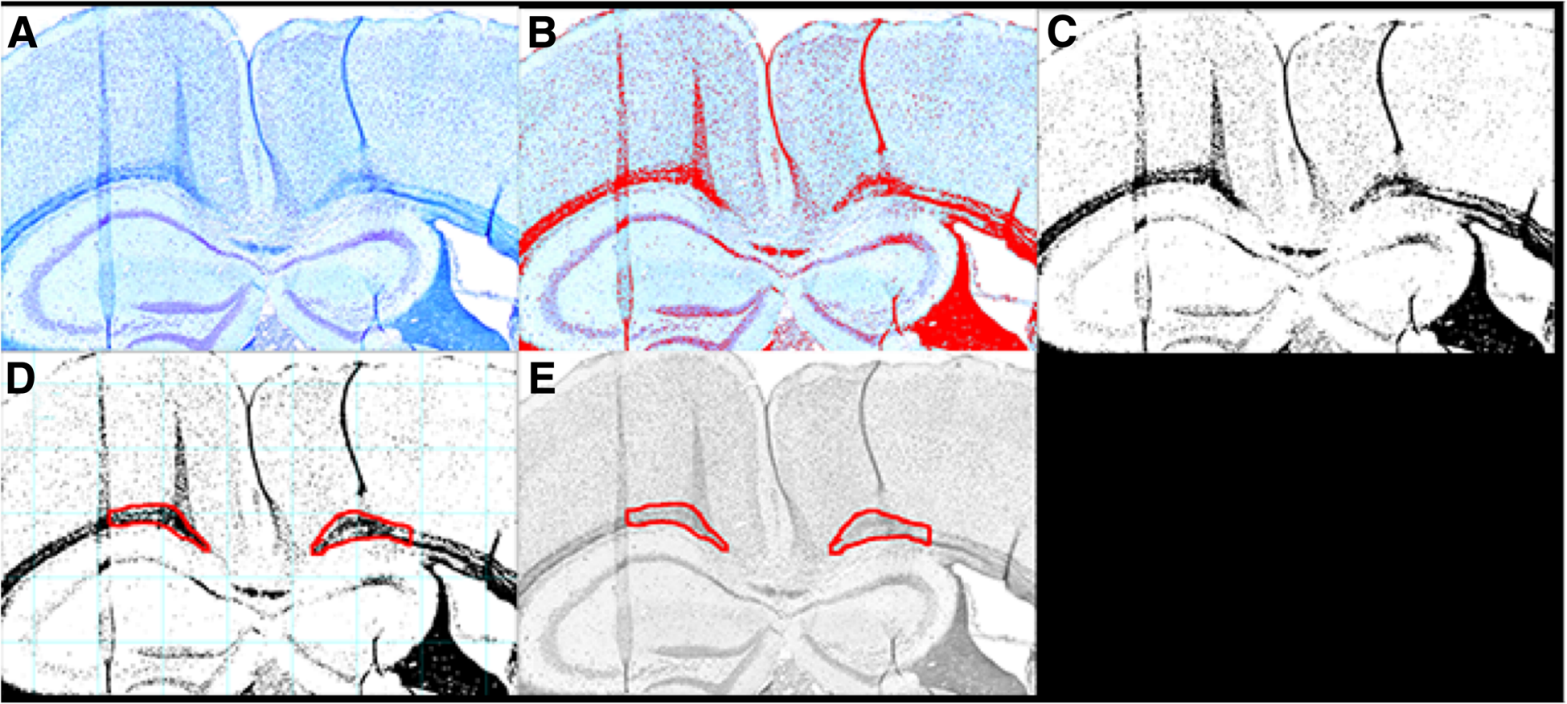
Analysis method of histology images of myelin. **a** Original color brightfield image. Note that the left portion of these brain images correspond to the left hemisphere of the mouse brain. **b** Color thresholding (shown in red) on the same image. Thresholding was done in color since saturation for myelinated tissues showed variation relative to unmyelinated regions. **c** Resulting black and white mask derived from (**b**). **d** Black and white mask with grid and both regions of interest outlined in red. Note that the left outline does not extend past the second tissue fold (anomaly), and that the length of the right outline matches that of the left, by design. **e** 8-bit black and white original image with both ROIs overlaid in red. We quantify the grey values indicative of myelin from this image, with darker hence more grey pixels associated with more myelin

All analysis was done using the Fiji version of ImageJ [27] – Fig. 4. Myelin-stained images were loaded into ImageJ, then thresholded in color, and scaled globally to achieve 0.32 pixels/μm. Next, a black and white mask of the image was created from the thresholded image. Myelinated areas within the corpus callosum region of interest were then outlined as follows. These outlines were done as conservatively as possible using the lasso tool: if an anomaly occurred on one side, outlines were extended to but not exceeding the anomaly, and the length of the outline on the contralateral side was measured to not exceed the maximum length of the constrained side. After outlining, the original image was converted to 8-bit greyscale, and both outlined ROIs were overlaid on top. Using the ROI manager in ImageJ, the measure functionality was used to determine the area, mean grey value, and maximum and minimum grey values from the greyscale image with the ROI overlays.

Pixel darkness was compared (left [ultrasound] versus right [control]) within a single image using the original, unmodified mean grey values from the ROI overlays in the ImageJ analysis. From this, a percent difference in myelin content was derived by calculating the difference between the average gray value for the left side minus that of the right side, divided by gray value from the right (control) side.

### Pilot then final study with MS mice

We performed a screening study with separate cohorts of demyelinating and remyelinating MS mice to facilitate qualitative histopathological analysis of the myelin content in the brain tissue samples associated with each ultrasound protocol (each one, again defined by the following carrier frequencies: 0.625 MHz, 1.09 MHz and 2.0 MHz) – six sets of mice, each with *n* = 4 mice/cohort. Through this qualitative analysis we identified the ultrasound protocol that had the greatest effect on myelin content for either or both of demyelinating and remyelinating states, then performed additional studies for that protocol on another three MS mice. We then pooled the histological results for that most promising ultrasound protocol applied to that demyelinating or remyelinating state, and performed the quantitative histological analysis described above on those seven mice.

## Results

### MRI scans

**Fig. 5:**
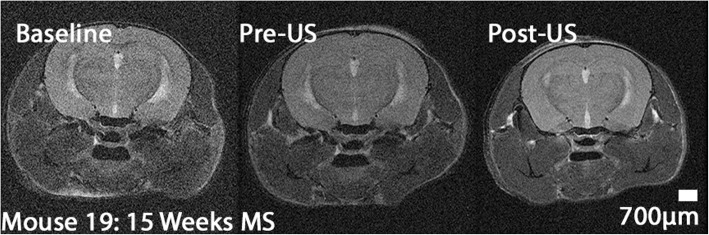
MRI imaging of long protocol (15 week) mice. This image demonstrates the presence of MS-like pathology, specifically decreased myelin in the corpus callosum, relative to their baseline images taken before introduction of Cuprizone, for a representative mouse brain exposed to 1.09 MHz. Note that the left portion of a given MRI image corresponds to the left hemisphere of that mouse brain

Short, 5 week protocol animals did not show significant change between baseline and pre-US MRI scans, consistent with model behavior and our decision to apply ultrasound before completion of demyelination (images not shown). Long, 15 week protocol animals showed significant demyelination during the remyelination phase in MRI images (Fig. 5) without, however, measurable changes in myelin content after ultrasound application (analysis not shown).

### EEG analysis

**Fig. 6:**
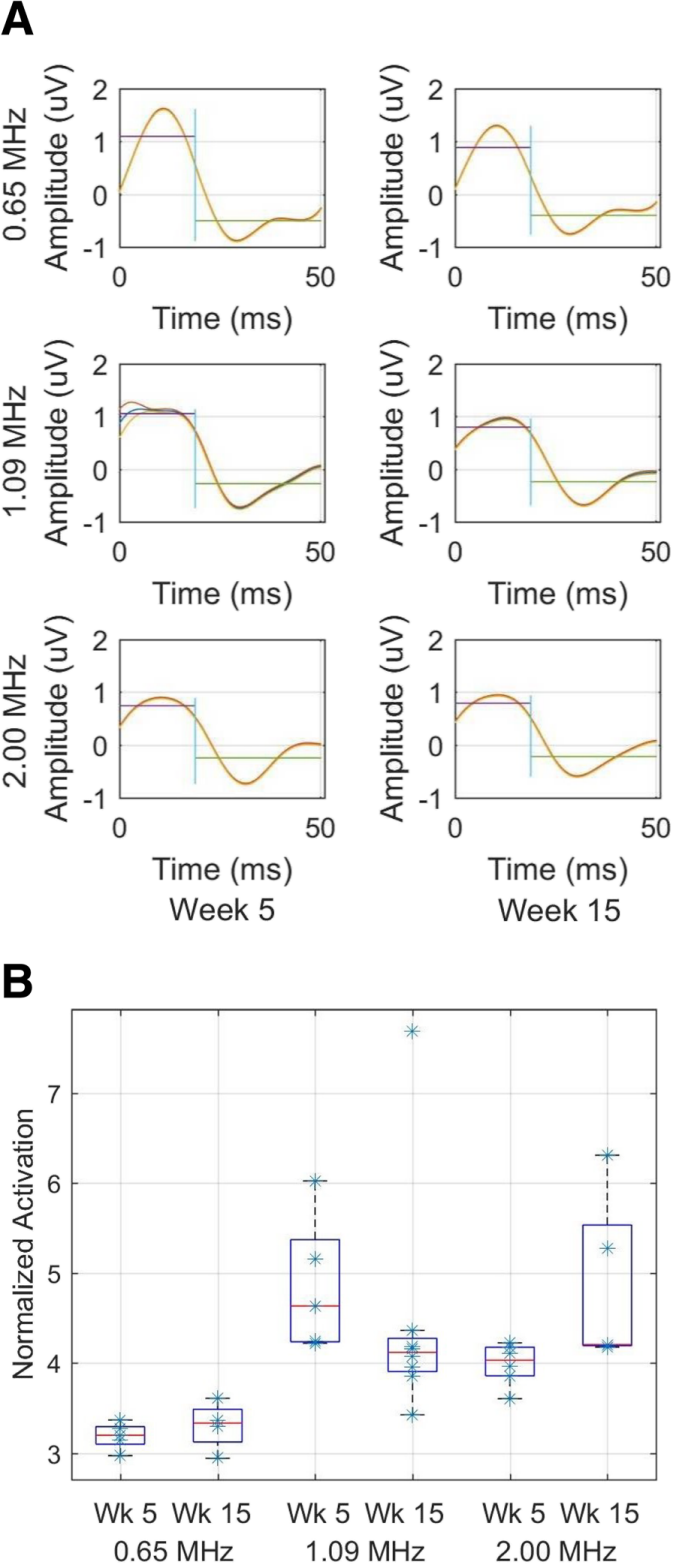
Ultrasound applied to MS-model mice successfully activates brain. **a** Mean and standard error of EEG signals recorded from a representative mouse within each MS and ultrasound protocol. Horizontal lines denote either the mean value of the EEG signal during ultrasound application, hence observed brain activation (the first 19 ms) or the mean value of the EEG signal during the time without ultrasound (the ‘background’, during the subsequent 31 ms). **b** Calculated for every 50-ms pair in the time series for each mouse, and aggregated across all mice for each protocol, this figure’s boxplots show the difference between mean brain activity induced by ultrasound and mean background brain activity, normalized by the absolute value of the mean background brain activity. (0.625 MHz, *n* = 4; 1.09 MHz, *n* = 4; 2.0 MHz, *n* = 4)

Both demyelinating (short, 5 week) and remyelinating (long, 15 week) studies demonstrated successful production of brain activation by ultrasound (Fig. 6a). In all cases, the mean amplitude recorded by EEG during ultrasound application was higher than that of the background. The two higher frequency protocols generated statistically significantly more brain activity than the low-frequency protocol for each of the 5-week and 15-week animals (Fig. 6b).

### Histological analysis

**Fig. 7:**
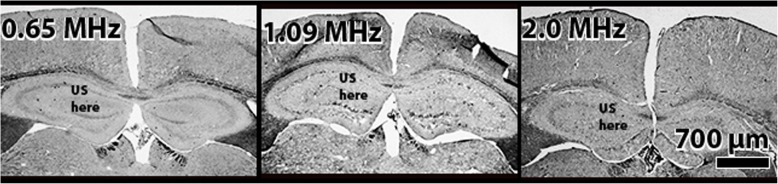
Representative coronal slices of MS mouse brain stained for myelin for each ultrasound protocol, designated by the frequency of ultrasound. Note that the corpus callosum for only the 1.09 MHz sample shows, qualitatively, enhanced myelin at the region of ultrasound application (above the annotation ‘US here’). Note that the left portion of a given brain image corresponds to the left hemisphere of that mouse brain

Visual inspection of histological slides stained for myelin for all (*n* = 4) short protocol therapeutic animals indicated no difference between pFU therapy and control sides of the brain (images not shown). Visual inspection of all (*n* = 4) long, 15-week MS mice told a different story – see Fig. 7. Note that these representative results for 0.625 MHz, and 2.0 MHz ultrasound protocol animals show no qualitative difference in myelin content between treated and untreated hemispheres, consistent with all the brain-tissue samples. However, visual inspection of our initial cohort of remyelinating MS mice exposed to 1.09 MHz ultrasound suggested that these animals had more myelin within the hemisphere of brain where ultrasound was applied relative to the contralateral side.

**Fig. 8:**
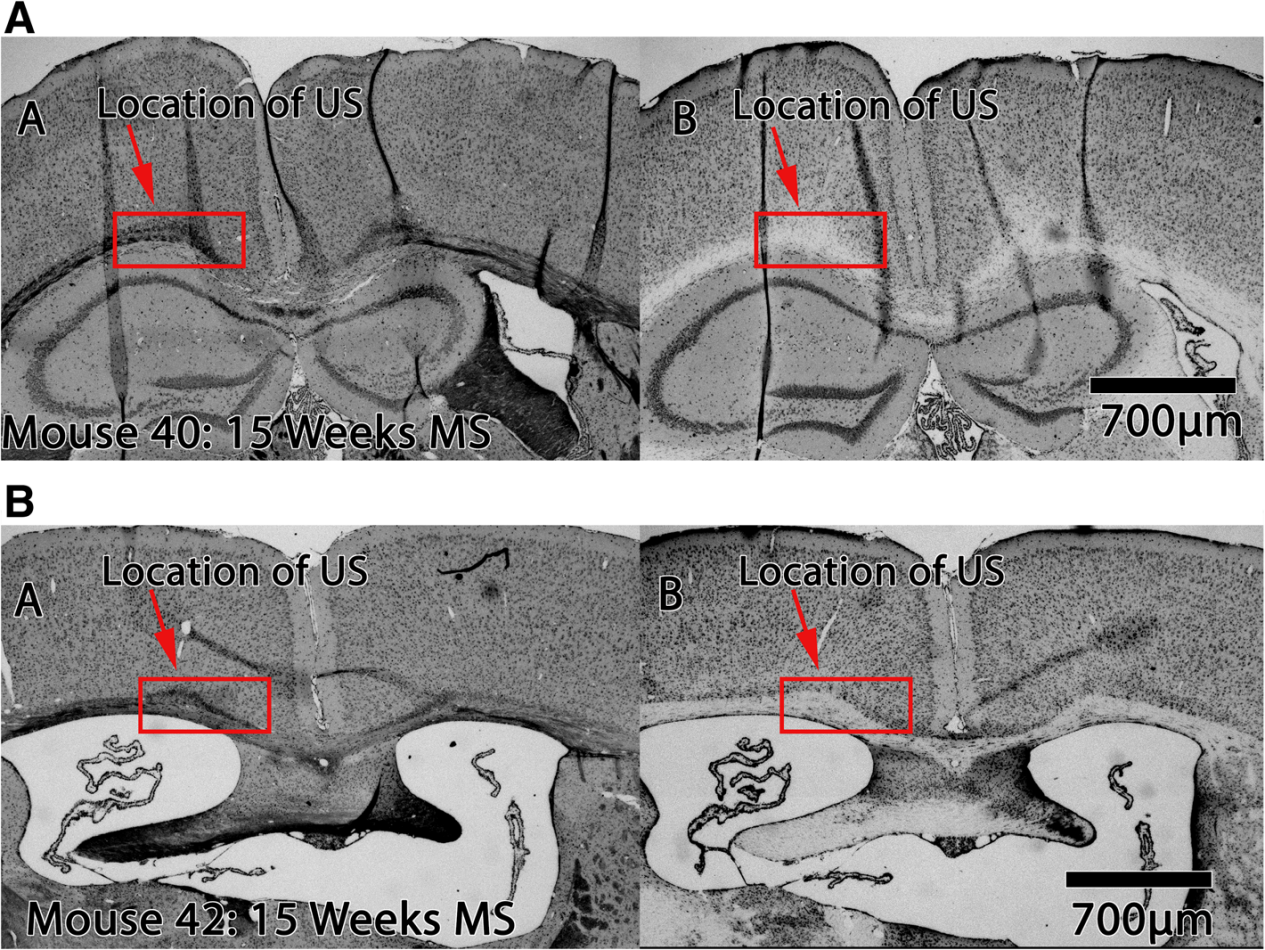
Myelin and Cresyl violet stains of representative mouse brains. (A,A’) Myelin stain of brain from representative 1.09 MHz long-protocol animals during remyelination studies. (B,B’) Cresyl violet stain of adjacent slices of brain from the same animals as in (A,A’). Note that the left portion of a given brain image corresponds to the left hemisphere of that mouse brain

Motivated by this promising qualitative result, we performed additional 15-week MS mouse studies (*n* = 3) using 1.09 MHz ultrasound, aggregated those new histological data with our initial findings, and then performed a quantitative analysis of the histology. In addition to more myelin (e.g., Figs. 7b and 8a and a’), we did not observe any neuronal damage from ultrasound application in any of the 7 animals at 1.09 MHz (e.g., Fig. 8b and b’).

**Fig. 9:**
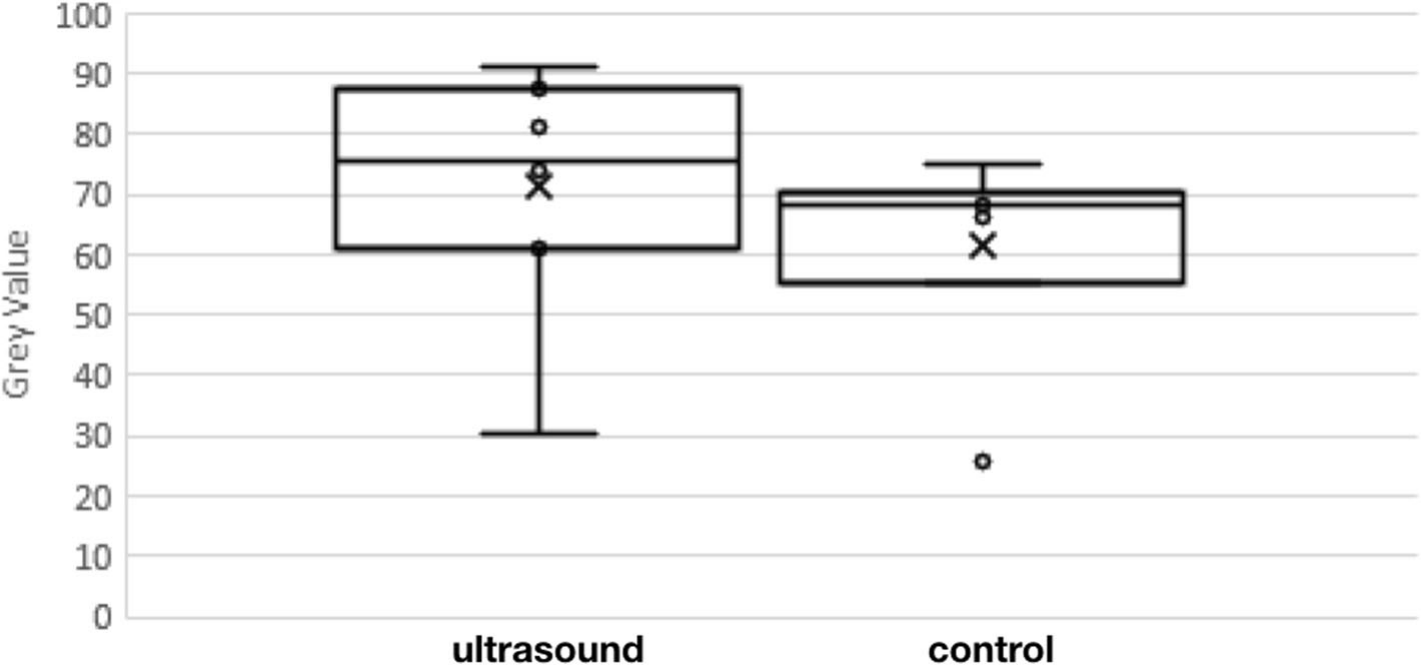
Gray-scale value that measures extent of myelin on the left side of mouse brain to which ultrasound was applied compared to that of the right (control) side. Note higher values denote more myelin. This difference in myelin between brain hemispheres had statistical significance (*n* = 7; two-sided, paired Student’s t-test, *p* < 0.02) with a substantial effect size (t score = 3.02)

Analyzing over all remyelinating mice exposed to 1.09 MHz, Fig. 9 and Table 1 together show the result of quantitative analysis of the amount of myelin associated with this ultrasound protocol, demonstrating that the side of brain exposed to ultrasound had more myelin than the control side, with statistical significance.

**Table 1:** Histological Analysis. Pixel darkness value for ipsilateral (ultrasound) vs contralateral (control) sides, with a higher value marking more myelin, with maximum value equaling 255

Animal Number	ipsilateral	contralateral	difference	% difference^a^	interpretation
Group 1					
1	30.6	25.7	4.9	19%	More myelin due to ultrasound
2	87.4	66.2	21.2	32%	More myelin due to ultrasound
3	90.9	68.5	22.4	33%	More myelin due to ultrasound
4	75.3	69.7	5.6	8%	More myelin due to ultrasound
Group 2					
5	61.2	55.2	6	11%	More myelin due to ultrasound
6	74	75	-1	-1%	More myelin away from ultrasound
7	81.3	70.2	11.1	16%	More myelin due to ultrasound
Mean (All Animals)	71.5+/−7.75	61.5+/− 6.39	10+/− 3.3	16.6+/− 4.7%	Net more myelin due to ultrasound

^a^ “% difference” = difference between ipsilateral and contralateral myelin values divided by contralateral value then expressed as a percent

## Discussion

Gibson et al. observed that an optogenetic stimulatory paradigm could cause myelin to thicken around central neurons made amenable to light-based stimulation thanks to transgenic techniques. Others have observed activation of brain function created by transcranially delivered ultrasound. Taken together, these results motivated the present study, which sought to ascertain whether or not ultrasound could activate demyelinated and/or remyelinating brain and thereby enhance the amount of myelin in those brains. To do so, we translated the temporal pattern of the optically-based neuron stimulation protocol of Gibson et al. into an ultrasound-based brain activation protocol. Through this exploratory work we identified one ultrasound protocol that accelerated remyelination, as demonstrated with histological analysis. In addition, this protocol did not damage mouse brain, as also shown with histological analysis.

Why did we observe accelerated remyelination in the hemisphere of brain that received neuro-stimulatory ultrasound, but did not observe decelerated demyelination? Perhaps ultrasound’s positive effect on myelin production can only have a net impact without the presence of the driving force for demyelination, here the cuprizone chow. Further work, with mouse models of MS based upon experimentally produced autoimmune disorder, itself treated or not treated during the study, represents a natural next step to explore this question as well as move this new therapeutic paradigm forward towards possible clinical tests.

## Limitations

As just discussed, we used a food-based model of multiple sclerosis, rather than one based upon lesion development created by an errant autoimmune system [28], the latter consistent with the human condition [29]. Future work will make use of such a model, with and without adjunctive therapy to fight the demyelination. Also, we used the portion of brain contralateral to ultrasound application as the control, leaving open the possibility that we may have activated the entire brain, hence increased myelin content on each side of the corpus collosum, though more efficiently ipsilateral to ultrasound application. Future studies with the autoimmune model will include a broader class of controls. Finally, we did not monitor for potential changes in behavior and function, the ultimate arbiters for success [30]. We intend to do so in the future.

Human skulls attenuate ultrasound much more than mouse skulls, and vary in structure both within a given human and between them. If, after further study, the 1.09 MHz ultrasound continues to provide optimal therapeutic benefit, ultrasound delivery systems will have to compensate for thicker, and variable-thickness human skulls, a need met by existing systems [21, 22]. We have speculated above that ultrasound alone may not suffice to remyelinate brain due to the on-going autoimmune attack central to multiple sclerosis, that it’s successful use may therefore require adjunctive anti-autoimmune treatment. Together, ultrasound and that adjunctive treatment may thus require chronic application, an expensive prospect given currently the high cost of MRI systems and their use. Future studies with an autoimmune mouse model must therefore involve study of chronic application of ultrasound and medication.

## Conclusion

Multiple sclerosis can severely impact patients, without, unfortunately, sufficient means of treating this disease. We have demonstrated in a mouse model of MS that focused ultrasound, known to activate brain, accelerated remyelination of MS lesions. MRI can readily identify MS lesions and MRI-guided focused ultrasound systems exist that can, in principle, deliver the ultrasound protocol we successfully tested here. Given the existence of these devices and the relatively low intensity value of our efficacious ultrasound protocol – 1.2 W/cm^2^, close to FDA limits for diagnostic ultrasound – we anticipate that future success with this approach to MS therapy using more realistic MS mouse models may 1 day translate rapidly to clinical trials that help address this devastating disease.

## Abbreviations

EEG: electroencephalography
FDA: food and drug administration
I_spba_: spatial peak burst average intensity values
I_sppa_: spatial peak pulse average intensity values
I_spta_: spatial peak temporal average intensity values
MHz: megahertz
MRI: magnetic resonance imaging
ms: milliseconds
MS: multiple sclerosis
pFU: pulsed focused ultrasound
PRF: pulse repetition frequency
